# IMU-Based Movement Trajectory Heatmaps for Human Activity Recognition

**DOI:** 10.3390/s20247179

**Published:** 2020-12-15

**Authors:** Orhan Konak, Pit Wegner, Bert Arnrich

**Affiliations:** Digital Health Center, Hasso Plattner Institute, University of Potsdam, 14482 Potsdam, Germany; pit.wegner@student.hpi.de

**Keywords:** human activity recognition, image processing, machine learning, sensor data

## Abstract

Recent trends in ubiquitous computing have led to a proliferation of studies that focus on human activity recognition (HAR) utilizing inertial sensor data that consist of acceleration, orientation and angular velocity. However, the performances of such approaches are limited by the amount of annotated training data, especially in fields where annotating data is highly time-consuming and requires specialized professionals, such as in healthcare. In image classification, this limitation has been mitigated by powerful oversampling techniques such as data augmentation. Using this technique, this work evaluates to what extent transforming inertial sensor data into movement trajectories and into 2D heatmap images can be advantageous for HAR when data are scarce. A convolutional long short-term memory (ConvLSTM) network that incorporates spatiotemporal correlations was used to classify the heatmap images. Evaluation was carried out on Deep Inertial Poser (DIP), a known dataset composed of inertial sensor data. The results obtained suggest that for datasets with large numbers of subjects, using state-of-the-art methods remains the best alternative. However, a performance advantage was achieved for small datasets, which is usually the case in healthcare. Moreover, movement trajectories provide a visual representation of human activities, which can help researchers to better interpret and analyze motion patterns.

## 1. Introduction

Human activity recognition (HAR) has many advantages in various disciplines, such as assistive technology. Prediction of human activities could play a pivotal role in healthcare’s future to check patient compliance with recommendations regarding physical activity or to investigate the causes of activity behavior. Indeed, a growing body of literature recognizes the importance of HAR [1,2,3]. As stated by Vrigkas et al. [2], this field can be categorized according to the nature of sensor data employed. Methods being used to perform activity classification highly depend on the type of sensor in action, e.g., cameras or motion-sensing sensors, each with both advantages and disadvantages. While cameras, for instance, can capture wide-ranging information, their use is prohibitive in some instances, e.g., in personal and professional spaces. This is a challenging issue that arises, especially in the healthcare domain. In contrast, sensors employing accelerometers and gyroscopes on several axes, also known as inertial measurement units (IMUs), are bound to the person wearing them [4]. Activity recognition using inertial sensors is attained through acceleration, orientation and angular velocity on three spatial axes [4]. Thanks to the expansion of smart devices and the possibility of embedding sensors into portable wearables and workwear, IMUs are ever more present and can be used almost without restrictions [5]. Moreover, the possibility of keeping track of activities indoors and outdoors continuously while protecting the user’s privacy has increased the popularity of IMUs in HAR [6,7,8,9,10].

However, a limitation of IMUs is the comparatively limited information content they provide compared to images from cameras, especially when it comes to HAR. In other words, nuances that can be captured by an image can hardly be reflected in IMU readings. The performance of IMU-based HAR is further constrained by the scarcity of annotated datasets, an issue of particular relevance when it comes to healthcare, given the high costs involved in annotating these datasets with highly skilled personnel. At the same time, image classification techniques such as data augmentation (DA) techniques and deep convolutional neural networks (CNNs) pre-trained on extensive datasets have proven more reliable and robust in comparison to the ones commonly applied to other sensor modalities [11].

**Figure 1:**
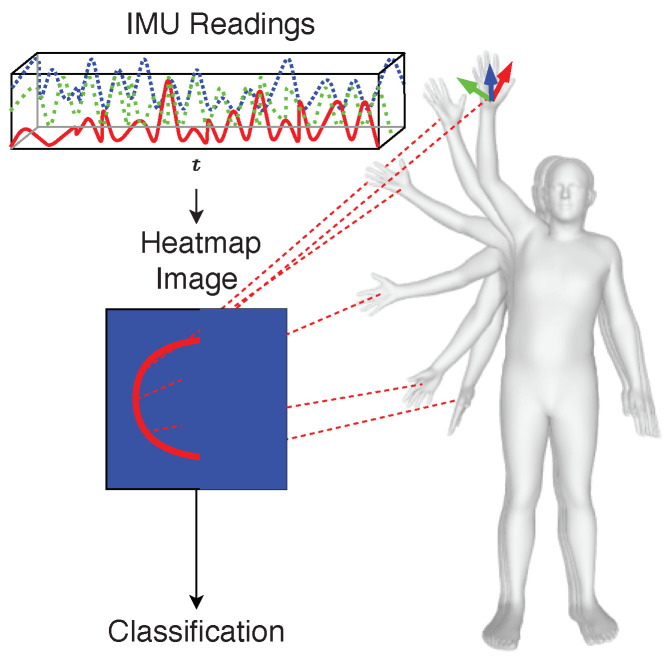
Transforming motion sensor data inputs into corresponding trajectories for classifying different activity types.

We hypothesize that applying well-known image processing techniques to IMU data can help address data scarcity while safeguarding subject privacy. This could enable researchers to take advantage of privacy protection and continuous monitoring from the IMUs and use a mature research field, such as image classification, potentially leading to new insights into movement patterns and better classification performance. To test this hypothesis, we needed a general method for obtaining movement trajectories from IMU readings. There have been approaches to generating trajectories from a single IMU, but this is still only possible for the lower body parts [12]. Building upon previous work by Huang et al. [13], we developed a new procedure to generate movement trajectories, i.e., positions over time, for any body part. These trajectories are then reduced into 2D heatmaps to facilitate computer vision data augmentation and classification techniques. The  heatmaps are then used as a proxy for the IMU readings. The overall approach is illustrated in Figure 1.

To evaluate the discriminative performance of the movement trajectory heatmaps in comparison to raw IMU data, we utilized DIP-IMU, the largest IMU dataset publicly available [13]. The experimental set-up entailed different long short-term memory (LSTM) configurations, including data augmentation. The results obtained suggest that the use of movement trajectories does present a performance advantage over established methods, but only for studies with small numbers of subjects. In addition, the generated images allowed a form of differentiation and interpretation that is more accessible to humans. As such, this work shows that sensor-based activity recognition can build upon an already existing field like image classification. As such, the main contributions of this work are two-fold, (1) a novel procedure to selectively track the trajectory of any body part using IMUs and further transform it into an image for activity recognition, and (2) evaluation of the usefulness of this transformation for HAR in data-scarce settings where privacy also plays an important role.

The remainder of this work proceeds as follows: In Section 2, the work is placed in the context of previous research on sensor modality transformation. Section 3 describes the methodology used to transform the raw motion data into 2D images and classify them into different activities. Section 4 presents the research findings and limitations, focusing on comparing the proposed method with state-of-the-art solutions on inertial sensor data and further discussed in Section 5. Lastly, in Section 6, we draw the conclusions on this work.

## 2. Related Work

Studies that rely on generating a visual representation from sensor data in the context of HAR can be categorized in three main approaches: (1) frequency spectrum transformations, (2) sensor footprint derivation and (3) IMU trajectory for indoor localization and pose estimation.

With respect to frequency spectrum transformations, Laput et al. [14] proposed a method for fine-grained hand activity recognition based on acceleration data obtained from a commodity-based smartwatch. The method relied on swapping the domain from 1D acceleration data to 2D images of time-frequency-spectral features. The classification was achieved through a variant of the VGG16 CNN architecture [15]. They demonstrated a 95.2% classification accuracy over 25 atomic hand activities of 12 people. Similarly, Jiang et al. [16] and Ravi et al. [17] applied a short-time discrete Fourier transform (STDFT) to time-series signals for constructing a time-frequency-spectral image. A CNN network was then applied to the image to recognize simple daily activities, such as walking and standing. The generated images are a visual way of representing the spectrum of frequencies of a signal as it varies over time. In these works, even though the performance was high, this approach would likely fail to recognize activities executed at the same pace, i.e., frequency, but with different movement patterns. Conversely, different people might execute the same activity, but at a different pace, i.e., a different spectral pattern. As such, the ability of this approach to capture complex movement patterns is limited.

When it comes to sensor footprints, Singh et al. [18] also introduced the concept of transforming a non-visually interpretable problem domain to a visual domain to leverage the effectiveness of pre-trained CNNs on visual data. Data were extracted from a pressure sensor to identify a person from the footprints of individual steps among 13 people. The proposed method’s classification performance, with an average identification rate of 78.41%, suggests that the system with a pre-trained CNN as a feature extractor can achieve higher accuracy rates than similar raw sensor signal methods. However, this approach relies on non-trivial sensors, such as floor surface pressure mapping, not IMUs. Such an approach is also limited in the range of activities that could be reliably recognized, since it only covers one body part, i.e., foot movements. Still, this work demonstrates the potential of swapping domains to change the HAR task into an image classification.

More explicitly addressing IMU trajectory generation, current works have not analyzed HAR directly, but rather used it for the sensor-derived trajectories to track indoor localization in a given space, e.g., a building, when GPS is not available. These approaches often rely on placing the IMUs on a lower body part to enable leveraging the concept of zero-velocity update [19]. Only recently, this approach was also extended to include the IMU sensors placed on the upper body (chest) for indoor localization [20]. However, these approaches have only limited applicability for HAR, since they ultimately rely on subject displacement, i.e., positional change in space. As such, any activities that do not involve a change of position in space would not be recognized.

Therefore, an approach is necessary to identify the limbs and/or other body parts’ positions, i.e., poses, and how they change over time regardless of the subject’s spatial positioning. For this, the essential requirement is being able to estimate such poses. Baldi et al. were able to develop an estimator for upper-body pose using IMUs [21]. Huang et al. developed a whole-body pose estimator with a combination of different IMUs [13]. Leveraging Huang et al.’s fully body pose estimator, which makes it possible to capture a wide range of body poses, our approach derives 2D heatmaps by tracking single positions and how they change over time. Therefore, our approach can then be used to recognize complex activities regardless of the body part and/or combination.

To the best of our knowledge, this is the first work that evaluates the concept of transforming IMUs-generated signals into trajectories for any given body part and into additional 2D heatmap images to exploit the sophistication of image classification techniques.

## 3. Methods

**Figure 2:**
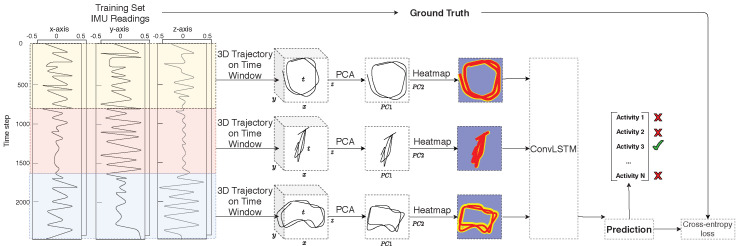
Overview: *Left:* implementation pipeline of modality transformation from 1D IMU data for given time windows into 3D trajectories. *Middle:* generating 2D heatmap images from 3D trajectories via deep inertial poser. *Right:* classification with ConvLSTM.

Our method can best be described as a series of data processing steps in a pipeline, starting from data collection and resulting in an activity classification. The necessary steps are detailed in Algorithm 1 and also shown in Figure 2.
**Algorithm 1:** IMU-based movement trajectory heatmaps for human activity recognition.
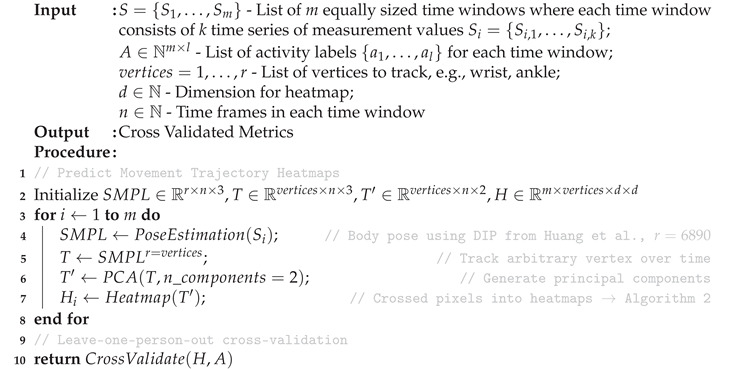


The pipeline describes a function f:S→A, where *S* is the set of time series coming from the IMU sensors and *A* the activity labels, such that $f(S_{i})$ is as close as possible to the actual activity performed. To generate the mapping *f* from IMU sensor time series data to images, the raw sensor data (acceleration and orientation) are first transformed to the global reference frame and then normalized to a local state-of-the-art statistical model called the skinned multi-person linear model (SMPL) [22]. The standardized raw data are then processed by a method proposed by Huang et al. called deep inertial poser (DIP), where six IMU signals on the wrists, knees, head and lower back are used to estimate a full human body pose [13]. Specific vertices of the hip-rooted 3D model striking the pose are tracked over time to form trajectories in 3D space. The windowed trajectories are flattened using principal component analysis (PCA) before being fed into the convolutional long short-term memory (ConvLSTM) model responsible for automatic feature extraction and classification. The entire pipeline comprises four key stages: data collection, pose estimation, movement trajectory heatmap generation and classification. A more detailed description for each step is provided in the following subsections.

### 3.1. Experimental Setup

To examine the performance of the proposed approach, both IMU readings and the corresponding position over time for an arbitrary point are necessary. Therefore, the number of available datasets is limited. One dataset, which includes both and was recorded for the purpose of pose estimation, is DIP-IMU [13]. The dataset contains approximately 90 min of real IMU data consisting of 10 (9 male, 1 female) subjects wearing 17 IMUs (Xsens sensors) for validation in 64 sequences with 330,000 time instants. Participants were asked to repeatedly carry out motions in five different categories, including controlled motion of the extremities (arms, legs); locomotion; and more natural full-body activities (e.g., jumping jacks, walking). The motion categories include, in total, 13 different activities. Table 1 provides an overview of the different activities.

The proposed approach uses the pre-trained model of DIP. Hence, the sensor setup closely resembles theirs. Six IMU sensors are placed at the locations of the wrists, knees, head and lower back. They each measure three-axis acceleration and global orientation. Using the measured values of a known initial calibration pose, the measured accelerations are cleaned of gravity (Equation (1)) and the measured rotation is set into relation to the calibration and virtual bone orientation (Equation (2)).

(1) $$\begin{array}{cc} a_{noG}\left(t\right) & =R_{IMU}\left(t\right)\cdot a_{IMU}\left(t\right)-g\\ a_{local}\left(t\right) & =R_{calib}\cdot a_{noG}\left(t\right) \end{array}$$

(2) $$R_{local}\left(t\right)=R_{calib}\cdot R_{IMU}\left(t\right)\cdot R_{bone}$$

As a final step, the accelerations and joint rotations are normalized with respect to the pelvis sensor location as the body root (Equation (3)), because it is the most stable location of the body and also acts as the root of the SMPL body model.

(3) $$\begin{array}{cc} a_{normalized}\left(t\right) & =R_{root}^{-1}\cdot \left(a_{local}\left(t\right)-a_{root}\right)\\ R_{normalized}\left(t\right) & =R_{root}^{-1}\cdot R_{local}\left(t\right) \end{array}$$

### 3.2. Pose Estimation

Generating trajectories from acceleration signals has been studied thoroughly, especially for gait analysis [23]. A reoccurring problem with sensors is the varying levels of noise that influence measurement reliability. Theoretically, calculating the position of an arbitrary point $x_{t}$ provided the acceleration of that point $a_{t}$ is possible by numerically integrating the signal twice, as seen in Equation (4). The variable $v_{t}$ denotes the velocity at time *t*. However, even a constant error from noise leads to a linear error in velocity and a resulting position’s quadratic error. Hence, the error is unbounded and drifts increasingly over time, shadowing the actual signal. In gait analysis, the problem can be circumvented due to the periodic nature of these motions. They include short periods of zero foot velocity when the foot is in contact with the ground. This pattern allows for precise drift error correction. Thus, merely relative position changes from the previous step need to be calculated through double integration of drift corrected accelerometer data. However, this does not apply for arbitrary motions in arbitrary sensor locations. Therefore, an indirect procedure is necessary to generate trajectories.

(4) $$x_{t+\Delta t}=x_{t}+\Delta t\left(v_{t}+\Delta t\left(a_{t+\Delta t}\right)\right)$$

**Figure 3:**
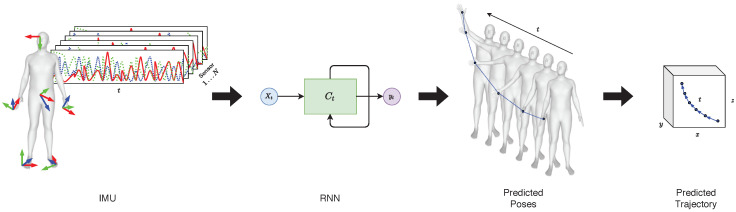
Overview: *Left:* IMU signals from sensors placed on a human body. *Middle:* a RNN takes the IMU signals as input and predicts a full body pose from only 6 sensors. *Right:* the algorithm allows selective tracking for each body part; hence it allows a point-wise trajectory generation.

The idea is to reconstruct a 3D human pose in each time step and then generate trajectories from it, as shown in Figure 3. In this work, we utilized DIP to estimate a full human body pose. The body’s shape and pose are described through the SMPL model. SMPL is a realistic 3D model of the human body that is based on skinning and blend shapes. The model can be expressed in the form $M\left(\beta ,\theta ;\Phi \right):R^{\left|\theta |\times |\beta \right|}\to R^{3r}$, which maps 10 shape parameters β and 72 pose θ parameters to vertices represented by a vector of length r=6890. The model, M(β,θ;Φ), is then

$$\begin{array}{cc} M(\beta ,\theta ) & =W(T(\beta ,\theta ),J(\beta ),\theta ,W)\\ T(\beta ,\theta ) & =T_{\mu }+B_{s}\left(\beta \right)+B_{p}\left(\theta \right) \end{array}$$

where *W* is a linear blend–skinning function, *J* a function to predict joint locations, W a set of blend weights, $B_{s}\left(\beta \right)$ a blend shape function, $B_{p}\left(\theta \right)$ a pose-dependent blend shape function and $T_{\mu }$ the base pose [22].

Given the model and a training dataset $D={\left(x^{i},y^{i}\right)}_{i=1}^{n}$, the mapping function h:x→y predicts the SMPL pose parameters given the IMU inputs (acceleration and orientation). Huang et al. [13] have shown on different datasets that the mapping performs best using bidirectional recurrent neural networks (BiRNN) with LSTM cells [24]. Experiments have shown that accurate real-time pose estimation can be generated with 5 future and 20 past frames resulting in a latency of only 85 ms [25]. Predicting the SMPL model enables tracking an arbitrary point over time.

### 3.3. Movement Trajectory Heatmaps

From the SMPL body model, virtually any vertex of the 6890 vertices of the 3D model can be chosen and tracked over numerous frames. The model allows for immense flexibility, despite having only six sensors for the initial data acquisition. The 3D coordinates of each frame are saved as an n-dimensional time series for each vertex location. For further processing as images, these trajectories are split into chunks of equal length to avoid extensive overlapping. The window size needs to be adjusted to the motion’s nature, minimizing overlap while conserving prominent patterns. In practice, time frames of 2.5–5 s were used.

The generated trajectories were recorded in a three-dimensional space in a sliding-window fashion. As motions are subtle with respect to the 2D image plane, we reduced the image’s dimensionality from 3D to 2D by applying PCA. The outcomes of this were sparse images.

Creating a trajectory image like that addresses the spatial domain, but neglects temporal relations, which might contain useful information. Hence, pixels crossed more often by the trajectory were counted and highlighted in heatmaps, as outlined in Algorithm 2.
**Algorithm 2:** Heatmap generation.
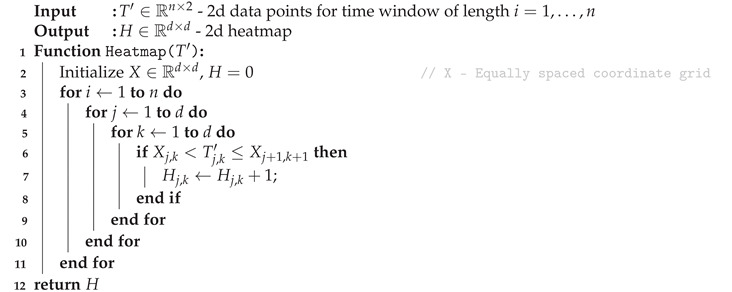


### 3.4. Classification

There are two main approaches to neural networks that are appropriate for time series classification and that have been demonstrated to perform well on activity recognition using wearable inertial sensors [26]: CNN and RNN models. They have also proven to be very useful in challenging computer vision problems when trained at scale for classifying objects in images. For sequential tasks, it may be more common to use an LSTM in conjunction with a CNN in a CNN-LSTM model or ConvLSTM model [27], because LSTM models alone are used to leverage the capability of memory cells to store information for later use, which is rather useful for time series prediction than classification. In such a case, a CNN model is used to extract features from a subsequence of raw sample data. An LSTM then interprets the outcome in aggregate [28].

For the presented approach’s comparison purposes, three different models were trained to solve the activity classification problem. The first model is trained directly on the raw sensor data sequences using LSTM. The second model classifies the same sequences using one-dimensional ConvLSTM. The third model extends the second model by taking a sequence of trajectory images of fixed-length time windows after dimensionality reduction as inputs to a two-dimensional ConvLSTM. The models were trained on TensorFlow-GPU with Keras as the high-level API [29]. Training was performed using the early stopping method to prevent overfitting the training data.

The network input format depends on the architecture in use. Three different formats were used: (1) unmodified sensor data of size 300×60 corresponding to 5 s of recording for each time window; (2) SMPL model pose parameters of size 300×216; and (3) the sequence of generated images using a window size of 150, resulting in an input size of 4×6×64×64. The designed LSTM model has two LSTM hidden layers, followed by a fully connected layer to interpret the features extracted by the LSTM hidden layer before the final output layer that was used to make the predictions. The image ConvLSTM model was composed of two consecutive ConvLSTM layers with 16 5×5 and 32 3×3 filters respectively; Maxpooling layers of size 2; and successive time distributed flattening and dense layers to interpret the extracted features over multiple time steps. As Keras does not provide a one-dimensional ConvLSTM layer, a very similar architecture was built manually. The output of each network is a dense softmax layer representing probabilities for each of the activity classes.

The models were trained using the efficient Adam version of stochastic gradient descent to optimize the network [30], and the categorical cross-entropy loss function was used given that this is a multi-class classification problem. There was only one element in the label vector, which was not zero, resulting in a loss function of

(5) $$L=-log\left(\frac{e^{s_{p}}}{\sum_{j}^{C}e^{s_{j}}}\right)$$

where *C* denotes the set of classes, *s* the vector of predictions of the network and $s_{p}$ the prediction for the target class.

## 4. Results

**Figure 4:**
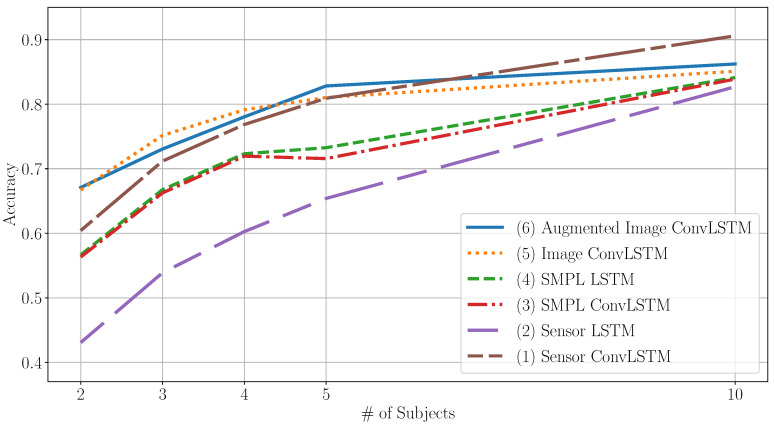
Model performance on DIP-IMU data over varying numbers of subjects on all 6 IMU signal inputs.

For the validation of the proposed method, we performed experiments using DIP-IMU. To capture the performance power of the proposed approach against reference methods in the field of sensor-based HAR, the following models were drawn for comparison: (1) ConvLSTM and (2) LSTM on the sensor signal; (3) ConvLSTM and (4) LSTM on the SMPL model; ConvLSTM on the generated images (5) without and (6) with DA. The accuracy for the considered cases is plotted in Figure 4 over subsets of the data with different sizes. The leave-one-person-out cross-validation evaluation scheme was adopted to evaluate the approaches.

The results show that the ConvLSTM model using heatmap images as inputs consistently achieves higher accuracy levels than LSTM on the raw sensor signal. To account for bias from the SMPL feature extraction, another LSTM model based on the SMPL body parameters was trained, yielding accuracy below the proposed ConvLSTM model using heatmap images. Looking at the results of the one-dimensional ConvLSTM on the raw sensor signal, training on the full dataset yields the highest classification accuracy of 90.6% but underperforms image ConvLSTM for smaller subsets of subjects.

One benefit of taking a detour off the generated heatmap images from trajectories is the possibility of image manipulation to virtually increase the amount of data available. The effects of the application of DA to the DIP-IMU dataset such as flipping and rotating, do not significantly benefit us in terms of absolute accuracy. However, it tends to have smaller confidence intervals for its predictions, promising more stable results, as can be seen from the data in Table 2.

**Figure 5:**
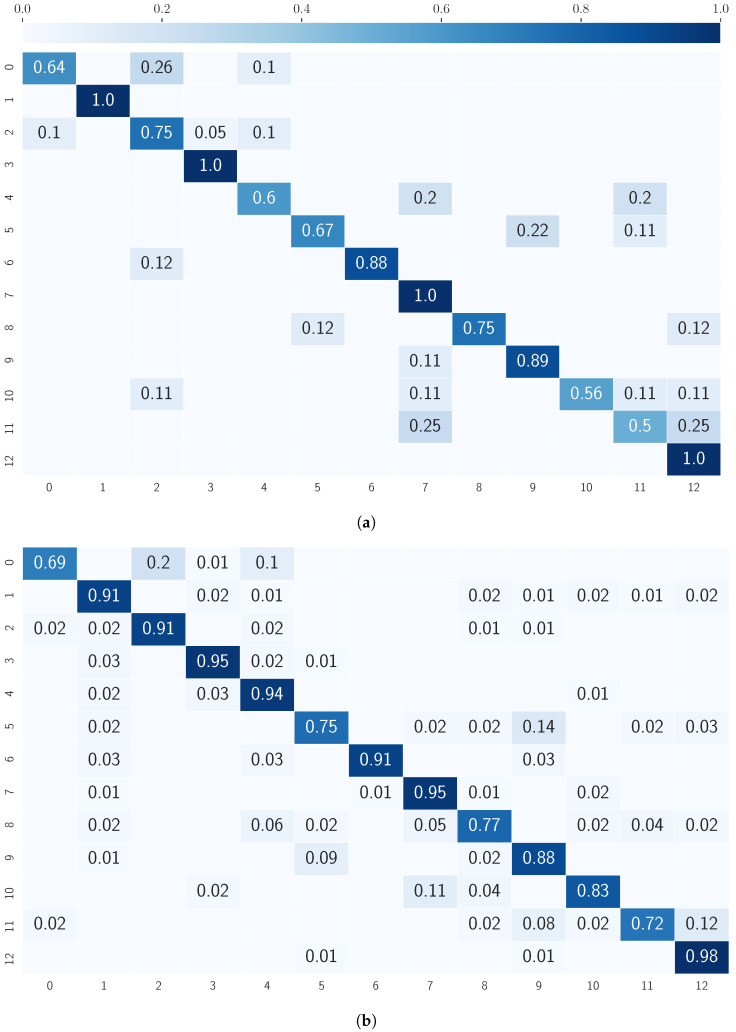
Normalized confusion matrix of activity recognition for the whole dataset with (**a**) ConvLSTM model on raw sensor signal and (**b**) ConvLSTM model on images (rows: ground-truth classes; columns: estimated classes). No value listed equals 0. Action list: (0) arm chest crossings; (1) arm circles; (2) arm head crossings; (3) arm raises; (4) arm stretches up; (5) cross stepping; (6) jumping jacks; (7) leg raises; (8) lunges; (9) side stepping; (10) squats; (11) sumo squats; and (12) walking.

By taking a closer look at the prediction results of the models in Figure 5a,b, certain patterns can be identified. In general, all models have difficulties predicting certain individual classes. For example, the “cross stepping” activity is one of the worst-performing for both approaches, most often confused with “side stepping”, a very similar motion. Additionally, “squats” and “sumo squats” are often confused with “leg raises” by the image ConvLSTM model. Another interesting pattern is that detecting “arm chest crossing” seems to work badly on both ConvLSTM models compared to the sensor LSTM model. Despite the similarity to the “arm head crossing” activity, the raw sensor data LSTM predicts this activity with 90% accuracy, whereas ConvLSTM predicts at least 20% of the samples incorrectly. The next chapter moves on to discuss these findings.

## 5. Discussion

This study assessed the importance of trajectory image classification over the direct classification of motion sensor signals. The most prominent finding to emerge from the analysis is that image ConvLSTM performs best on a smaller subset of subjects but then plateaus, while ConvLSTM on the raw sensor signal rises steadily for more subjects. The finding implies that the pose prediction as a preprocessing step reduces noise and extracts features allowing for early generalization. As the number of data increases, complex models learn to generalize better from self-learned features. The lower asymptote of the presented method is likely due to missing information that gets lost within the pose prediction, such as global coordinate system reference and rooting the model at the hip.

The outcome also implies that the predicted poses could be the reason for achieving higher accuracy for small subject numbers. Therefore, both an LSTM and a one-dimensional ConvLSTM model based on the SMPL body pose parameters were trained, which yielded worse accuracy rates in comparison with the ConvLSTM model on the heatmap images, but performed better than the raw sensor-based LSTM. This suggests that both pose estimation and ConvLSTM classification of images contribute to the final result independently of each other.

**Figure 6:**
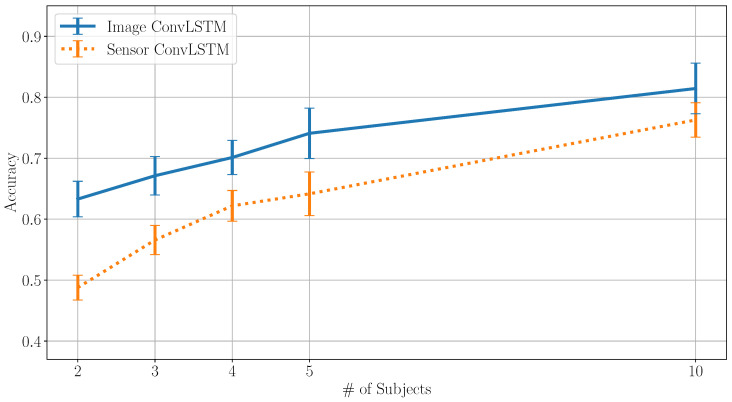
Model performance on DIP-IMU data for a varying number of subjects on 1 IMU signal input.

To evaluate the method on a smaller sensor setup, for example, a single smartwatch, another set of models was trained using only the right wrist sensor as an input. In the process, the SMPL pose prediction failed to accurately predict body limb positioning, leading to uncharacteristic trajectories and poor classification results. Both models trained on the raw sensor data outperformed the ConvLSTM image classification significantly. However, a perfect sensor, simulated by using the predicted pose of all six sensors and then only feeding the right wrist trajectories, could achieve better results for the ConvLSTM image classification compared to the raw sensor data. The results are set out in Figure 6. On the other hand, this may also be due to the fact that the information encoded in the right wrist movements of the body model includes influences of the other sensors introduced by the DIP network.

The confusion of some activities like “squats” and “sumo squats” with “leg raises” by the image ConvLSTM model was likely due to the hip-rooting of the SMPL body model, resulting in similar leg raising motions for each of the three activities. Contrary to expectations, the classification accuracy for “arm chest crossing” with LSTM on sensor data was much higher as compared to ConvLSTM for both input types, sensor data and images. This discrepancy could be attributed to the higher spatial correlations of similar movements, such as “arm head crossings”, which is discriminated by the convolutional layers, whereas LSTM predictions are more suited to capture more long-range information. Other common confusions mainly allude to similar movements of body parts during the different activities, activity overlap inside one sample window and pauses between the activities.

**Figure 7:**
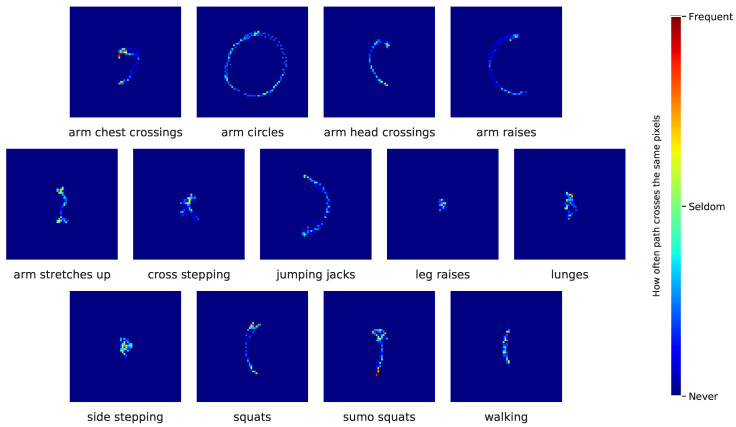
Examples of heatmaps for a generated trajectory of the right wrist for each class of activity.

Overall, these results indicate that images are more accessible for human interpretation of the achieved results. For example, taking a closer look at the images generated for the activities “arm circles” and “arm raises”, displayed in Figure 7, which are just a small subsample of the whole sequence on the right wrist, the values of the confusion matrix in Figure 5a indicate good performance. Additionally, the human eye can perceive more detail and make a better distinction between the different movements. On the other hand, images of the activities “squats” and “sumo squats”, which did not perform well, are also confusing for the human eye, since the movements seem slight variations in a planar representation. While IMU-based time series data leave no room for interpretation, motion-based trajectory images are well suited for explaining particular classification behavior.

### Limitations

The proposed method is mainly limited by the complex sensor setup that it requires. As of now, image classification with only one sensor is insufficient for most tasks. All six sensors have to be applied in a specific order to achieve acceptable and competitive prediction accuracy, even for single handed activities. Permanently wearing six IMU sensors (e.g., sewed into workwear) requires substantially more planning and resources than merely wearing a smartwatch or carrying a smartphone. Furthermore, slightly different sensor setups or using sensors from a different manufacturer could require extensive transfer learning from the original synthetic Archive of Motion Capture as Surface Shapes (AMASS) dataset [31]. Gathering high-quality ground truth data using motion capture or a Kinect camera may not be feasible. Optical motion capture provides the most accurate ground truth; it requires a full camera and marker setup. A sensor with less noise and drift or a better pose estimator could help overcome this limitation in theory.

Another limitation of using trajectory images is the loss of the time domain. Using heatmaps adds frequency information to the location, yet direction and speed are lost from the original data. The influence of this limitation enormously varies with the type of activities that have to be classified.

Both an advantage and a disadvantage of the method is the ability to manually select the vertices on the SMPL body model for which the trajectories will be generated. The experiment presented in this work used the sensor locations as key intuitive points to track. However, this choice is arbitrary, and an optimal choice may not be intuitively guessed. The vertex choice should also consider the main body parts involved in the targeted activities.

## 6. Conclusions and Future Work

The current study’s main goal was to determine the classification power of human motion from images generated from IMU readings. The investigation showed that this data transformation improved HAR classification accuracy compared to the state-of-the-art methods for relatively small datasets. However, it fails to maintain competitive accuracy for more subjects in the wake of information loss during feature engineering. Overall, this study strengthens the idea of transforming data into a different research domain to use multidisciplinary techniques. The results contribute to the rapidly expanding field of digital health, where most of the studies conducted are still small regarding the number of subjects. Additionally, the types of possibly deployable devices are limited due to regulatory and privacy concerns. This is the first study that examines associations between IMU readings and corresponding trajectory images for HAR on any body part to the best of our knowledge. These findings contribute in several ways to our understanding of classification methodology and provide a basis for visual interpretation of motion.

Further research might explore the impacts of pre-trained models, such as the ones trained on the Modified National Institute of Standards and Technology (MNIST) handwritten dataset [32], which seems like a good fit compared to our generated images. Future research might also apply noise reduction techniques for IMUs to improve the signal-to-noise ratio, as suggested in [33]. This could help overcome the complex experimental setup. In addition, the optimal sensor location might prove an important area for future research. Beyond that, 3D image classification’s effectiveness instead of 2D images with concomitant loss of information could also be investigated in more depth. A hybrid model is recommended to ensure higher accuracy for both settings, fewer subjects and wealth information utilization with more subjects. Hence, two submodels for feature extraction in both strands, IMU and image, can be created to make full use of each classification strength.

## Figures and Tables

**Table 1:** Descriptions of activity labels.

Activity	Description
Arm Chest Crossings	Starting from T-pose, both arms are folded together against the chest and stretched out again
Arm Circles	either one or both arms are repeatedly circled around the shoulder forwards or backwards
Arm Head Crossings	Starting from T-pose, both hands are brought behind the head and stretched out again
Arm Raises	Starting from I-pose, one or both arms are repeatedly raised straight up to shoulder height and afterwards lowered
Arm Stretches up	Starting from a straight pose with hands up, the shoulders are touched and then reached up again
Cross Stepping	Walking sideways faced orthogonal to walking direction, each step in front of the other
Jumping Jacks	Repeated jumps involving legs spreading and arms stretching out and over the head
Leg Raises	One knee is repeatedly raised up to hip height and lowered again
Lunges	One big step with forward shifted weight is performed and then reversed
Side Stepping	Walking sideways faced orthogonal to walking direction, one foot catching up on the other
Squats	From a standing position, a sitting motion up to a knee angle of 90° is performed, before straightening up again
Sumos	Similar to squats, legs spread at a wide angle
Walking	Repeated forward steps on a straight line

**Table 2:** Accuracy comparison of methods on DIP-IMU dataset with all 10 subjects.

Input Modality		Accuracy
	Classifier	
Raw Sensor Data		
(1)	ConvLSTM	90.59 (±1.55)
(2)	LSTM	82.70 (±2.43)
SMPL Parameters		
(3)	ConvLSTM	83.89 (±2.24)
(4)	LSTM	84.12 (±2.01)
Trajectory Images		
(5)	ConvLSTM	85.11 (±8.29)
(6)	Augmented ConvLSTM	86.23 (±2.59)

## Code Availability

All source code and models are publicly available on GitHub https://github.com/hpi-dhc/trace-imu under MIT license.
